# A New Pathway Promotes Adaptation of Human Glioblastoma Cells to Glucose Starvation

**DOI:** 10.3390/cells9051249

**Published:** 2020-05-18

**Authors:** Alberto Azzalin, Francesca Brambilla, Eloisa Arbustini, Katia Basello, Attilio Speciani, Pierluigi Mauri, Paola Bezzi, Lorenzo Magrassi

**Affiliations:** 1 Neurosurgery, Dipartimento di Scienze Clinico-Chirurgiche e Pediatriche, Università degli Studi di Pavia, Fondazione IRCCS Policlinico S. Matteo, 27100 Pavia, Italy; azzalin.alberto@gmail.com; 2 Dipartimento di Biologia e Biotecnologie, University of Pavia, 27100 Pavia, Italy; 3 Proteomics and Metabolomics Institute for Biomedical Technologies (ITB-CNR), Segrate, 20090 Milan, Italy; francesca.brambilla@itb.cnr.it (F.B.); pierluigi.mauri@itb.cnr.it (P.M.); 4 Molecular Genetic Laboratory-Transplant Research Area, Fondazione IRCCS Policlinico S. Matteo, 27100 Pavia, Italy; E.Arbustini@smatteo.pv.it; 5 Cryolab, University of Rome Tor Vergata, 00133 Rome, Italy; katia.basello@gek-group.com (K.B.); attilio.speciani@gek-group.com (A.S.); 6 Inflammation Society, 18 Woodlands Park, Bexley, Kent DA52EL, UK; 7 Département des Neurosciences Fondamentales, Université de Lausanne, 1005 Lausanne, Switzerland; paola.bezzi@unil.ch; 8 Istituto di Genetica Molecolare-CNR, 27100 Pavia, Italy

**Keywords:** glioblastoma cells, aerobic glycolysis, GLUT/SLC2A, SHC3, PARP1

## Abstract

Adaptation of glioblastoma to caloric restriction induces compensatory changes in tumor metabolism that are incompletely known. Here we show that in human glioblastoma cells maintained in exhausted medium, SHC adaptor protein 3 (SHC3) increases due to down-regulation of SHC3 protein degradation. This effect is reversed by glucose addition and is not present in normal astrocytes. Increased SHC3 levels are associated to increased glucose uptake mediated by changes in membrane trafficking of glucose transporters of the solute carrier 2A superfamily (GLUT/SLC2A). We found that the effects on vesicle trafficking are mediated by SHC3 interactions with adaptor protein complex 1 and 2 (AP), BMP-2-inducible protein kinase and a fraction of poly ADP-ribose polymerase 1 (PARP1) associated to vesicles containing GLUT/SLC2As. In glioblastoma cells, PARP1 inhibitor veliparib mimics glucose starvation in enhancing glucose uptake. Furthermore, cytosol extracted from glioblastoma cells inhibits PARP1 enzymatic activity in vitro while immunodepletion of SHC3 from the cytosol significantly relieves this inhibition. The identification of a new pathway controlling glucose uptake in high grade gliomas represents an opportunity for repositioning existing drugs and designing new ones.

## 1. Introduction

Enhanced glucose metabolism in glioblastoma, as demonstrated in vivo by positron emission tomography, is associated with poor patient survival [1]. Hyperglycemia has an adverse prognostic impact in patients affected by glioblastoma [2,3] and high intracellular glucose increases resistance of glioblastoma cells to therapies targeting epidermal growth factor receptor, phosphoinositide 3-kinase and AKT [4]. Furthermore, in high-grade gliomas, enhanced glucose uptake through glucose transporters is important for brain tumor initiating cell growth and survival [5] and it is essential for survival of the mesenchymal subtype of glioblastoma [6]. Moreover, in glioblastoma, alternative metabolic pathways that use glutamine to compensate for the absence of glucose are poorly developed [7,8]. Maintaining an adequate level of glucose inside the cell is thus vital for glioblastoma cell growth and survival.

Glucose uptake in glioblastoma cells is tightly regulated and depends mainly on transporters of the GLUT/SLC2A superfamily [9,10,11]. Inhibition of the GLUT/SLC2As present on the membrane of glioblastoma cells reduces growth and enhances the effect of chemotherapy both in vitro and in vivo [11].

In glioblastoma as in many other tumors, intracellular glucose is metabolized through aerobic glycolysis [12] but the tricarboxylic acid cycle is also active [13]. Normal and aberrant growth factor receptors are modulating glycolysis through different pathways often leading to the activation of mammalian target of rapamycin complex 2 (mTORC2) [14]. The activation of mTORC2 complex by inducing post-translational modifications of forkhead box O (FoxO), relieves suppression of c-Myc by multiple microRNA networks [15]. Activation of focal adhesion kinase (FAK) by integrins may also modulate glucose entry and enhance aerobic glycolysis through mTORC-dependent [16] and -independent pathways [17]. We have recently shown that growing glioblastoma cells in monolayers at high cell density or in tridimensional spheroids leads to FAK activation and increases the intracellular levels of the two isoforms of SHC3: p52SHC3 and p64SHC3 [18]. SHC3 is a multifunctional docking protein characterized by two phospho-tyrosine binding sites linked by a proline-rich intermediate region [19] whose increase is correlated in vitro and in vivo with high-grade glioma and ependymoma survival [20,21]. SHC3 shares over 80% of sequence homology with Shc1, a protein involved in insulin/IGF1 signaling [22,23]. Mice lacking p66Shc1 have low levels of all other Shc1 isoforms and display increased insulin sensitivity and glucose tolerance [24] with reduced glycolysis and increased gluconeogenesis in muscle and liver [23,25].

We now show that culture conditions that increase the level of SHC3 in high-grade glioma cells enhance aerobic glycolysis, as demonstrated by increased glucose consumption and lactate production. Conversely, reduction of SHC3 levels by RNA interference or the use of dominant negative variants of SHC3 reduces glucose uptake and glycolysis. These effects are correlated to an increase in glucose uptake due to changes in vesicle trafficking resulting in more GLUT/SLC2As at the level of the membrane. We also found that depletion of glucose in the culture medium increases the level of SHC3. In order to study the mechanism of SHC3 modulation of glucose uptake, we characterized by mass spectrometry multiple proteins involved in vesicle assembly and turn-over that co-immunoprecipitated with SHC3. Our findings indicate that SHC3 interacts with AP1 and AP2 complexes that are associated with vesicle traffic at the plasma membrane and between the trans-Golgi network and the endosomes [26]. Moreover, we found that the portion of SHC3 that is purified in the same vesicular fraction containing GLUT/SLC2As is also associated to PARP1. These results demonstrate that glioblastoma cells can exploit multiple pathways to increase glucose uptake in order to maintain the high glycolytic activity that is typical of these ominous tumors.

## 2. Materials and Methods

All cell lines, vectors and reagents employed are listed in the key resource table (Table S1).

### 2.1. Cell Cultures, Vectors and Transfection Protocol

U-87 MG, Hu-197, C6 and HeLa cells were grown in DMEM/F12 (Thermo Fisher Scientific, Waltham, MA, USA) supplemented with 10% FBS, 100 unit/mL penicillin, 0.1 mg/mL streptomycin and 8 μg/mL ciprofloxacin and passaged once they were reaching confluence. Different culture conditions were introduced according to the experimental requirements and are specified where appropriate.

Primary human glioblastoma cultures were obtained from anonymized human glioblastoma specimens obtained in the course of previous studies as described [18,27]. After in vitro expansion for one passage the cells were frozen in multiple aliquots in the presence of FCS and DMSO and then maintained in liquid nitrogen up to the time of thawing for use in the experiments here described. As stated in the original publications, glioblastoma samples were obtained in accordance with local, national and international ethical guidelines and in full agreement with the principles of the Declaration of Helsinki and its revisions.

All plasmids were transfected according to a published polyethylenimmine (Sigma-Aldrich, St. Louis, MI, USA) based protocol [28]. Cells used for transfection were grown in six-well plates. To each well we added 5 μg of plasmid DNA pre-complexed with different amounts of PEI (1M, pH 7.0, Sigma-Aldrich, St. Louis, MI, USA) depending on the cell line transfected: U-87 MG 0.7 μL, Hu197 0.5 μL and HeLa cells 1.5 μL.

Plasmids containing either the entire human p52SHC3 cDNA or only the SHC3 PTB domain that, when expressed, has a dominant negative effect on SHC3, were previously described [23]. Plasmids encoding the SHC3-targeting shRNA inserted into pGeneClip hMGFP were obtained from Qiagen (Hilden, Germany). Vectors expressing eGFP fused either to the carboxy-terminal part of GLUT4 or the amino-terminal part of GLUT3 were generous gifts of Dr. Lauritzen [29] and Dr. Greenlee [30], respectively. Vectors expressing GFP-clathrin light chain and GFP-AP2 were generous gifts of Dr. Wu [31].

### 2.2. Western Blot

After the appropriate experiments cells were harvested, centrifuged and the pellet lysed in ice-cold RIPA buffer (150 mM NaCl, 50 mM Tris-HCl pH 8.0, 1% TritonX-100, 0.1% sodium deoxycholate, 0.1% sodium dodecyl sulphate, 1 mM Na_2_VO_4_ supplemented with Complete Mini Proteases Inhibitor Cocktail (Roche, Basel, Switzerland). Total proteins were quantified by Qubit protein assay kit (Thermo Fisher Scientific, Waltham, MA, USA) according to the manufacturer’s protocol. We added to the desired amount of protein extracts an equal volume of 2× Laemmli sample buffer (2% SDS, 6% glycerol, 150 mM ß-mercaptoethanol, 0.02% bromophenol blue and 62.5 mM Tris-HCl pH 6.8) and denatured for 5 min at 96 °C before loading onto a 12% polyacrilamide gel. Proteins were then transferred onto nitro-cellulose membrane Protran (GE Healthcare Life Sciences, Chicago, IL, USA). Membranes were blocked with 5% non-fat milk in PBS containing 0.1% Tween 20 and incubated over-night at 4 °C with the proper primary antibody. After washing, incubation with the appropriate HRP-labeled secondary antibody and further washing (3×), we detected the bound antibodies with the “ECL Select Western Blotting Detection Kit” (GE Healthcare Life Sciences, Chicago, IL, USA) according to the manufacturer’s instructions.

### 2.3. Measure of Glucose Consumption/Uptake and Lactate Production

Glucose consumption and lactate release into the cell culture medium were measured at the appropriate times by collecting 300 μL of medium from the cultures; after centrifugation (200 RCF) to remove floating cells and cell debris, glucose and lactate were measured by an enzymatic (glucose oxidase or lactic oxidase, respectively) amperometric method on an automatic blood gas analyzer (ABL800 Flex, Radiometer, Milan, Italy) certified for clinical use.

Glucose uptake by glioblastoma cells was determined by measuring (1,2-^3^H)-DG or 2NBDG incorporation. Briefly, 3 × 10^5^ U-87 MG cells per well were plated in 6-wells plates and subjected to the appropriate treatments; at the end of the treatments 2NBDG was added to the cells at the final concentration of 30 μM in glucose free medium. At the appropriate time for the experiments the cells contained in at least three wells were washed in cold PBS and lysed in 200 μL of RIPA buffer. In each sample 2NBDG content was measured using a fluorometer (Qubit, Thermo Fisher Scientific, Waltham, MA, USA). Alternatively, after the appropriate treatments 2 mL of fresh medium containing (1,2-^3^H)-DG (specific activity 10Ci/mmol) were added to each well at a final concentration of 125nCi/mL. After 15 min of incubation the cells were washed four times in ice-cold PBS, detached by trypsin/EDTA digestion and after centrifugation (300 RCF) lysed in 0.5 mL 100 mM NaOH. Then we added 8 mL of Pico-Fluor plus liquid scintillation cocktail (PerkinElmer, Waltham, MA, USA) to the cell lysates. Cells were counted on a Z2 Beckman Coulter counter (Beckman Coulter, Brea, CA, USA).

### 2.4. Quantitative RT-PCR Analysis

Total RNA was extracted with TRIzol (Thermo Fisher Scientific, Waltham, MA, USA) according to the manufacturer’s instructions and quantified by Qubit fluorometer (Thermo Fisher Scientific, Waltham, MA, USA). One μg of total RNA was reverse-transcribed using the High Capacity cDNA Archive kit (Applied Biosystem, Foster City, CA, USA). Custom gene-specific TaqMan Minor Groove Binder (MGB) probes and primers sets for *SHC1, SHC3* and *ACTB* were from Thermo Fisher Scientific (Waltham, MA, USA). qPCR reactions (40 cycles, 95 °C 10 min and 59 °C 1 min) were performed on ABI PRISM 7900 HT platform (Applied Biosystems, Foster City, CA, USA). Amplifications were performed in 50 μL containing primers (900 nM each), probe (200 nM) and 1X “Universal PCR Master mix No Amperase UNG” (Thermo Fisher Scientific, Waltham, MA, USA). *ACTB* was used for normalization. Ct averages of the replicas performed for each gene were determined and the ∆Ct (Target gene Ct-*ACTB* Ct) was calculated for each sample; finally, for each gene the ∆∆Ct (∆Ct G^−^-∆Ct G^+^) was calculated.

### 2.5. Measure of SHC3 Protein Stability

**Figure 1:**
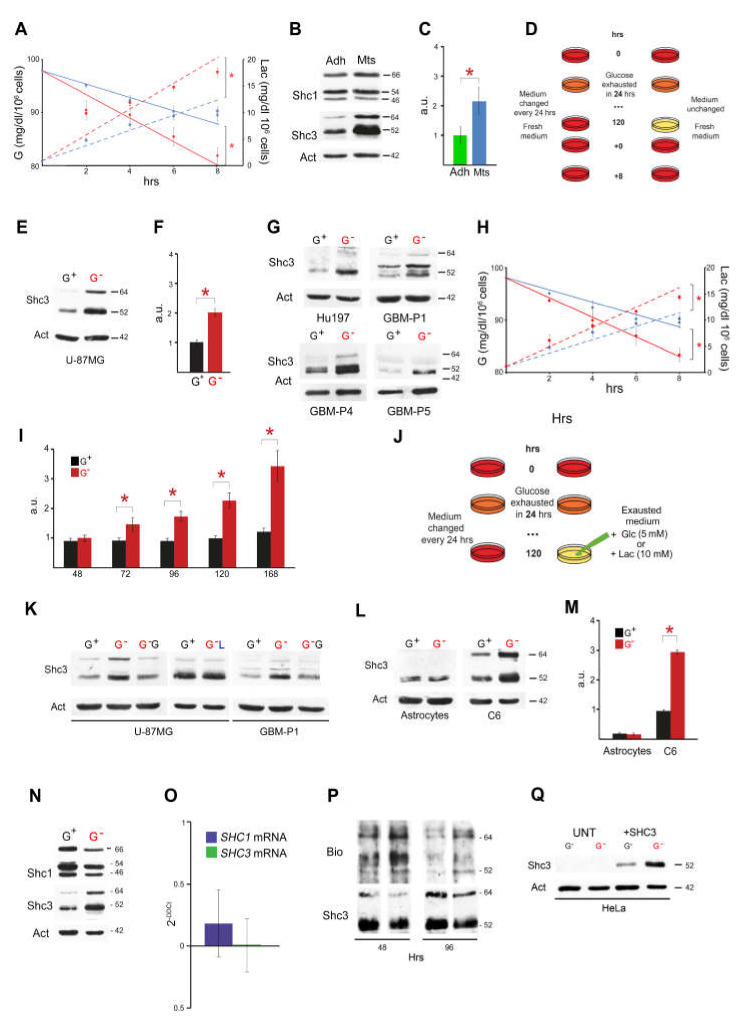
Rate of glucose uptake and SHC3 level increase in glioblastoma cells maintained in exhausted medium. (**A**) Rates of glucose consumption (left axis of ordinates) and lactate production (right axis of ordinates) in stable human glioblastoma cell line U-87 MG growing as MTS or in adherence. Cells growing as MTS (red) have elevated level of SHC3 and show an enhanced uptake of glucose and increased lactate production compared to adherent cells (blue). Here and in the following figures, linear regression of the data was used to outline the differences in glucose metabolism and lactate secretion between treatments without any implication for the true kinetic of the biochemical process. Data are means ± SD (at least n = 3 experiments, *= *P* < 0.05). (**B**) Western blot using protein lysates from U-87 MG growing in adherence (Adh) or as floating spheroid (MTS). Proteins indicated on the left were demonstrated after reaction with the appropriate antibodies, on the right molecular weights in kDa. (**C**) Densitometry analysis of differences in SHC3 levels in experiments similar to that shown in B. Data are means ± SD (n > 3 experiments; *: *P* < 0.05). (**D**) Schematic of the culture conditions for glioblastoma cells growing in adherence that enhance SHC3 levels and glucose uptake. (**E**) Western blot using protein lysates from stable human glioblastoma cell lines U-87 MG, cells maintained as indicated in D; G^+^ medium changed daily, G^−^ (red) medium unchanged for 5 days. Both p52SHC3 and p63SHC3 are increased in cells maintained in glucose spoiled medium. (**F**) Densitometry analysis of differences in SHC3 levels in experiments similar to that presented in E. Data are means ± SD (n > 3 experiments; *: *P* < 0.05). (**G**) Same as in E but with human glioblastoma cell line Hu197 and 3 different primary cultures, GBM-P1, P4 and P5, that were originally derived from the dissociation of glioblastoma samples. At least one of the isoforms of SHC3 increased in cells maintained in glucose spoiled medium (G^−^, red). Please notice than in GBM-P1 and GBM-P4 cells the band corresponding to p52SHC3 split in two close bands. This is a known phenomenon that depends in part on the extent of post-translational modifications. (**H**) Same as in A but here the U-87 MG cells were all growing in adherence according to D: medium replaced every 24 h (blue), medium not replaced (red). (**I**) Densitometric analysis, time course of p52SHC3 immunoreactivity using protein lysates of U-87 MG cells maintained with medium changed daily (G^+^) or left unchanged (G^−^, red). Data are means ± SD (at least n = 3 experiments, *: *P* < 0.05); a.u.: optical density in arbitrary units. (**J**) Schematic of the culture conditions for U-87 MG glioblastoma cells growing in adherence, glucose addition to the exhausted medium induced a rapid decrease of SHC3. (**K**) Western blot using protein lysates from stable human glioblastoma cell lines U-87 MG and primary human glioblastoma cells (GBM-P1) maintained as indicated in L with the addition, 8 h before harvesting, of glucose (G^−^G) or lactate (G^−^L) to reach a final concentration of 5 or 15 mM, respectively. Glucose addition, but not lactate addition, decreased the level of SHC3 to normal, suggesting that lack of glucose and no other molecules accumulating in the exhausted medium was mainly responsible for the increase in SHC3. (**L,M**) Western blot and densitometry analysis using protein lysates from normal rat astrocytes and C6 gliosarcoma cells. Glucose starvation does not induce SHC3 increase in normal rat astrocytes cultured without medium replacement (G^−^, red); on the contrary cells of the rat gliosarcoma cell line C6, like human glioblastoma cells, show increased SHC3 levels after growth in glucose exhausted medium. Abbreviations and data as in D and E. (**N**) Western blot of protein lysates of U-87 MG cells maintained in glucose containing (G^+^) or glucose free medium (G^−^). SHC3 levels in glucose deprived cells were increased, while levels of Shc1 did not changed significantly. (**O**) qPCR analysis of total mRNA extracted from the same cells as in A. Levels of SHC1 and SHC3 mRNAs did not significantly change in the cells in glucose free medium despite a significant increase in SHC3 protein level. (**P**) Western blot of proteins immunoprecipitated with an anti-SHC3 monoclonal antibody from U-87 MG cells pulse-labeled with AHA and then maintained as outlined in Figure 1D. AHA incorporated into the proteins was revealed by biotin conjugation using an alkyne-based reaction. Biotin-labeled SHC3 isoforms were revealed through reaction with avidin conjugated to horseradish-peroxidase and they resulted significantly higher in extracts from cells in glucose exhausted medium, suggesting that SHC3 undergoes a slower degradation in the absence of glucose. (**Q**) Western blot of protein lysates of HeLa cells maintained as shown in Figure 1D, after transfection of a plasmid encoding for p52SHC3 cDNA under an artificial housekeeping promoter that is not sensitive to glucose changes. The differential increase of SHC3 in HeLa cells maintained in absence of glucose is a further indication that glucose deprivation induces an increase in SHC3 through post-translational effects.

Nascent proteins were labeled in U-87 MG cells by the overnight addition of AHA (final concentration 2 mM) to DMEM medium without methionine and at the end of the incorporation the medium was removed. The cells were washed several times with fresh medium and then maintained according to the scheme shown in Figure 1D. Total proteins were extracted from cells growing in the presence or absence of glucose and SHC3 was immunoprecipitated from these extracts with anti-SHC3 monoclonal antibody linked to protein G paramagnetic beads (μMACS—Miltenyi Biotech, Bergisch Gladbach, Germany). After extensive washing and before elution of SHC3 from the beads, we reacted the azide of the AHA incorporated into the immunoprecipitated proteins with alkyne-biotin using the “Click-iT Protein Reaction Buffer Kit” (Thermo Fisher Scientific, Waltham, MA, USA) according to the manufacturer’s instructions. After extensive washing the proteins were eluted from the beads with sample buffer and subject to western blot analysis. The amount of biotin linked to the proteins was detected with HRP-conjugated streptavidin (Dako, Santa Clara, CA, USA) and “SuperSignal West Dura Extended Duration Substrate” (Thermo Fisher Scientific, Waltham, MA, USA).

### 2.6. Imaging Flow Cytometry Analysis

All imaging flow cytometry analysis (IFCA) were performed in living U-87 MG previously maintained in vitro either in the presence (G*+*) or absence of glucose (G^−^). Briefly: 10^6^ U-87 MG cells were scraped in ice cold PBS containing 3% BSA (PBSA), washed in the same solution by centrifugation (0.8 G) and incubated at 4 °C in a 1:20 dilution of mouse monoclonal anti-GLUT1 (Merck-Millipore, Burlington, MA, USA) in PBSA for 1 h. After 3× washing in the same solution without the primary antibody the cells were incubated for 30 min at 4 °C in a 1:20 dilution of goat anti-Mouse IgG (whole molecule)–R-Phycoerythrin-labelled (Sigma-Aldrich, St. Louis, MI, USA) in PBSA. After washing 3× by centrifuging and resuspending the cells in ice cold PBSA, we immediately proceeded to IFCA using an ImageStreamX MarkII flow cytometer (Amnis, Luminex Corporation, Austin, TX, USA). The experiment was repeated three times. Data were collected using Inspire software (Amnis, version 2.0) with the following parameters: 10,000 images per sample, 488 nm laser (25 mW and 100 mW) to excite the PE tagged secondary antibody, 785 nm laser used to provide a side scatter signal and measurement of SpeedBeads (Amnis, Luminex Corporation, Austin, TX, USA), 830 nm laser used for internal bead calibration of core flow speed and focus, 60× objective, in low speed flow. The gating strategy is better described in results. Data were further analyzed by Ideas software (Amnis, version 6.1). The logs of each Feature value (Area, Aspect Ratio, Intensity) are used to plot both fluorescence and scatter parameters. Intensity Feature analysis is measured within the default mask or the created Membrane mask. The Intensity Feature was defined as the sum of the background-subtracted pixel values within the masked area of the image. The Aspect Ratio Feature was the minor axis divided by the major axis, which describes how round or oblong an object is. The Area Feature is measured in microns squared, after which the number of pixels is converted to micrometers.

### 2.7. Transferrin Endocytosis

We measured vesicle recycling by transferrin endocytosis with and without blocking the recycling of biotinylated-transferrin (B-Tfn) [32,33,34,35,36]. U-87 MG cells were grown according to the scheme in Figure 1D; after 120 h of growth with or without medium replacements they were detached and their number determined by counting an aliquot on a Z2 Beckman Coulter counter (Beckman Coulter, Brea, CA, USA). The cells were resuspended into 7 mL of ice cold Optimem medium (Thermo Fisher Scientific, Waltham, MA, USA) supplemented with 1 g/L D-glucose and 500 ng/mL B-Tfn and maintained in ice for 15 min to saturate Tfn receptors present on the membrane while blocking vesicles endocytosis. Endocytosis was then restarted by moving the cell suspension to a 37 °C bath. In those experiments where we blocked the re-uptake of the endocytosed B-Tfn, we added unlabeled holo-transferrin (5 mg/mL) to the solution containing the cells, just before moving them to 37 °C [33]. After 0, 1, 2, 5 and 7 min we mixed 0.5 mL of the cell suspension with 1 mL of pre-cooled acidic buffer (0.2 M acetic acid, 0.5 M NaCl, pH 2.5) in order to remove all the B-Tfn still bound to the cell membrane and transferred the aliquots on ice for 10 min. One further aliquot of the cell suspension was collected at time 0 (T0) and processed as described but without the addition of the acid buffer in order to measure the B-Tfn initially bound to the membrane, a value that reflects the concentration of transferrin receptors present on the membrane. After acidic treatment on ice we pelleted the cells contained in each aliquot by centrifugation (300 RCF). The cell pellet was lysed by boiling in denaturing buffer and the cell lysate was subjected to polyacrilamide gel electrophoresis. After blotting, the amount of B-Tfn present was evaluated with HRP-conjugated streptavidin (Dako, Santa Clara, CA, USA) and “SuperSignal West Dura Extended Duration Substrate” (Thermo Fisher Scientific, Waltham, MA, USA). After densitometric analysis, the amount of incorporated Tfn for each treatment was normalized on its corresponding actin signal and this value was normalized to the total B-Tfn bound to the cell membrane. Finally, we subtracted to all values the B-Tfn signal obtained at t_0_ after acidic buffer treatment. This last value corresponds to the B-Tfn that not-specifically entered the cells before the 37 °C incubation [33].

### 2.8. Immunohistochemistry and TIRF

U-87 MG glioblastoma cells seeded on 24 mm-diameter coverslips were fixed on ice for 10 min with 4% paraformaldehyde in PBS. After repeated washes in PBS, they were incubated overnight at 4 °C with primary antibodies in PBS with the addition of 1% BSA and 0.4% saponin. Cells were then extensively washed in PBS, exposed to fluorescein-conjugated secondary antibodies in PBS and 1% BSA for 1 h at RT, washed again, incubated with DAPI for 5 min and finally mounted (FluorSave; Merck-Millipore, Burlington, MA, USA). Images were taken at RT in a laser confocal microscope (TCS SP2; Leica-Microsystems, Wetzlar, Germany). Confocal images were rendered using confocal software (LCS Lite; Leica-Microsystems, Wetzlar, Germany). Illustrations were prepared using Photoshop software (Adobe, San Jose, CA, USA).

For TIRF illumination imaging experiments, the expanded beam of a 488/568-nm argon/krypton multiline laser (20 mW; Laserphysics, UK), passed through a laser wavelength selector (AOTF; VisiTech International, UK ) synchronized with a charge-coupled device camera (SNAP-HQ; Roper Scientific, Sarasota, FL, USA) under control of Metafluor software (Universal Imaging, Bedford Hills, NY, USA), was introduced to the coverslip from the high NA objective lens (1.45 NA; α-Plan Fluar 100×; Carl Zeiss, Oberkochen, Germany). Light entered the coverslip and underwent total internal reflection at the glass–cell interface. In our experimental conditions, penetration depth of TIRF illumination was calculated to be 92 nm [37,38]. In the TIRF/EPI illumination imaging protocol used in our experiments, EPI was generated by a polychromator illumination system (excitation light of 488 nm; Visichrome; Visitron, Puchheim, Germany). Once the fluorescent protein expressing cell was recognized, TIRF illumination (excitation of 568 nm) was switched on. The pixel size was 126 nm. The dynamics of individual eGFP-GLUT3 or GLUT4 puncta was studied in TIRF experiments by monitoring the eGFP fluorescence in a 1.25-μm-diameter circle positioned on top of the spot and in a concentric annulus (inner diameter of 1.25 μm; outer diameter of 2.5 μm). Fusion events were recognized as the series of stereotyped fluorescence changes as illustrated before [37,38]. Just after the fusion event, the bright eGFP fluorescence spread to finally disappear. The temporal distribution of fusion events was obtained by plotting them against time.

### 2.9. Immunoprecipitation Assays

After the appropriate experiments, cells were lysed in ice-cold RIPA buffer (150 mM NaCl, 50 mM Tris-HCl pH 8.0, 1% TritonX-100, 0.1% sodium deoxycholate, 0.1% sodium dodecyl sulphate, 1 mM Na_3_VO_4_) supplemented with Complete Mini Proteases Inhibitor Cocktail (Roche, Basel, Switzerland). Cell lysates generally were mixed to micromagnetic beads conjugated to the appropriate antibody and left to react for 5 h at 4 °C with continuous rotation. Micromagnetic beads (“Dynabeads MyOne Carboxylic Acid”, Thermo Fisher Scientific, Waltham, MA, USA) covalently conjugated with the appropriate antibody were prepared following manufacturer’s instructions for “two step coating procedure”. Immunoprecipitated proteins were eluted from the antibody linked to the beads by denaturation in the same protein loading buffer used for Western blotting.

ECFP/SHC3 fusion proteins were immunoprecipitated using the “μMACS GFP Isolation Kit” (Miltenyi Biotec, Bergisch Gladbach, Germany) following manufacturer’s instructions.

### 2.10. LC-MS Analysis

1 × 10^7^ U-87 MG cells grown into 10 Petri dishes and subjected to the specified treatments were collected and lysed in RIPA buffer. CoIP was performed with magnetic beads covalently linked to SHC3, as described previously; elution was performed with “Pierce Elution Buffer” Thermo Fisher Scientific, Waltham, MA, USA) after elution samples were desalted on “Zeba Spin Desalting Columns, 7K MWCO” (Thermo Fisher Scientific, Waltham, MA, USA). After desalting, samples were directly subjected to in-solution tryptic digestion (Trypsin Gold, Promega, Madison, WI, USA), using a 1:50 trypsin/protein ratio, o/n at 37 °C, and 1:100 trypsin/protein ratio, 4 h at 37 °C. Digestion was terminated by addition of TFA to a final concentration of 0.5%; digested samples were stored frozen at −80 °C until use. Prior to the analysis by LC-MS, all samples were desalted and enriched using PepClean C18 columns (Pierce, Thermo Fisher Scientific, Waltham, MA, USA).

Each trypsin-digested CoIP sample was analyzed by liquid chromatography coupled to tandem mass spectrometry (LC-MS/MS). Briefly, 10 μL of peptide mixtures were on-line concentrated and desalted by C18 traps loaded on a 10-port valve, prior to final separation on the capillary reversed phase column (Biobasic-C18, 0.180 i.d. ×100 mm, 5 μm particle size, Thermo Fisher Scientific, Waltham, MA, USA). Peptides were separated with the following eluents: (A) 0.1% formic acid in water; (B) 0.1% formic acid in acetonitrile; the gradient profile was 5% eluent B for 5 min, 5–40% B in 45 min, 40–95% B in 15 min; flow rate on C-18 column was 1 μL/min. The peptides eluted from the C18 column were directly analyzed with an LTQ mass spectrometer (Thermo Fisher Scientific, Waltham, MA, USA) equipped with a nano-ESI source. Full MS spectra were acquired in positive mode over a 400−2000 *m*/*z* range, followed by five MS/MS events sequentially generated in a data-dependent manner on the first five most intense ions selected from the full MS spectrum (collision energy 35%) and using dynamic exclusion for MS/MS analysis.

Using the Bioworks 3.3.1 software, based on the SEQUEST algorithm (University of Washington, licensed to Thermo Fisher Scientific, Waltham, MA, USA), the experimental tandem mass spectra were correlated to theoretical peptide sequences obtained by in-silico digestion of the *Homo sapiens* protein database, downloaded from NCBI and UniPROT. Processing was done using a tolerance on the mass measurements of 2.00 amu for precursor peptide and 1.00 amu for product ions. In order to reduce false positive identifications, stringent filters were applied for peptide matching: minimum values of Xcorr ≥ 1.5, 2.0 and 2.5, respectively, for single, double and triple charge ions; peptide/protein probability ≤10−3 and consensus score ≥10. False discovery rate, calculated through a decoy database, was less than 5%. For protein identification, only the first best-matching peptide was taken into consideration. Only proteins identified by two or more MS/MS spectra were considered. Protein lists obtained by the SEQUEST algorithm were translated in non-redundant gene list and further elaborated using the Multidimensional Algorithm Protein Map (MAProMa) software for their alignment [39].

### 2.11. Immunoelectron Microscopy

After fractionation by ultracentrifugation, the pellet containing the LDM fraction was collected and fixed in Karnowsky’s solution (0.5% glutaraldehyde, 2% paraformaldehyde in 0.2 M cacodylate buffer, pH 7.3) for 2 h and post-fixed in 1.5% osmium tetroxide in the same buffer. The pellet was then dehydrated and embedded in Epon-Araldite resin according to standard protocols. Immuno-electron microscopy was carried out on ultra-thin sections mounted on parlodion-coated nickel grids. The sections on the grids were etched with 3% H_2_O_2_ for 10 min, treated with 5% sodium metaperyodate for 10 min at room temperature, enzymatically pre-digested with 0.5% trypsin in Tris buffer with 0.05% CaCl2 (15 min, 37 °C) and then rinsed in 0.05 M TRIS/HCl buffer, pH 7.4, and incubated in the same buffer with 1:20 normal goat serum for 30 min at room temperature. The grids were incubated overnight at 4 °C with the primary antibodies (mouse anti-SHC3 monoclonal Ab, dilution 1:10, and rabbit anti-PARP1 polyclonal antibody, dilution 1:10), rinsed in Tris buffer, re-incubated with two secondary antibodies both diluted 1:10, one against mouse IgG conjugated to 5 nm colloidal gold particles and the other against rabbit IgG conjugated to 15 nm colloidal gold particles. After repeated washing the sections were stained with uranyl acetate and lead citrate. The specificity of the immunoreactions was checked using normal sera as the primary antibodies. All grids were visualized using a Zeiss 902 electron microscope (Carl Zeiss, Oberkochen, Germany).

### 2.12. Cell Fractionation and Vesicles Separation

At the end of the appropriate treatments 5 × 10^7^ U-87 MG cells were harvested by trypsin/EDTA digestion. After trypsin inactivation with serum and 3 washes in serum free medium the cells were resuspended in the homogenization buffer (0.25 M sucrose, 1 mM EDTA, 20 mM Hepes-NaOH, pH 7.4) containing Complete Mini Proteases Inhibitors (Roche, Basel, Switzerland) and homogenized on ice in a 7 mL glass Dounce homogenizer. After pelleting the nuclei by centrifugation of the suspension for 10 min at 200 RCF, we pelleted organelles and high density microsomes by centrifuging the supernatant at 23700 RCF. Supernatants were finally centrifuged for 30 min at 234179 RCF to isolate low density microvesicles (LDM). Each cellular fraction was then lysed by RIPA buffer and subjected to western blot analysis. In addition, LDM were fixed for electron microscopy analysis. Finally, in fractionation experiments, LDM were suspended in the homogenization medium and iodixanol (60%, Visipaque, GE Healthcare Life Sciences, Chicago, IL, USA) to obtain a final iodixanol concentration of 14% or 30% (*w*/*v*). The samples were loaded into “Thinwall polypropylene tubes” (Beckman Coulter, Brea, CA, USA) and density gradients were formed at 264931 RCF in the SW55Ti rotor (Beckman Coulter, Brea, CA, USA) at 4 °C for 4 h. Serial 300 μL fractions were collected from top, quantified by Qubit and 10 μg total proteins were subjected to immunoblotting analysis.

### 2.13. In Vitro Inhibition of PARP-1 Activity

Human PARP1 activity in presence or absence of cytosolic extracts from Hu197 was measured in vitro using the HT Universal Colorimetric PARP Assay Kit with Histone-coated Strip Wells (Trevigen, Gaithersburg, MD, USA, Cat# 4677-096-K) according to the manufacturer’s instructions. Human glioblastoma Hu197 cells were grown according to the scheme in Figure 1D, 1.5 × 10^7^ cells grown in presence (G^+^) or absence of glucose (G^−^) were collected. After blocking trypsin with 10% FBS-containing medium, the cells were washed 3 times in PBS (4°). Cells were lysed by Dounce homogenization in hypotonic buffer (20 mM Hepes, 10 mM KCl, 2 mM MgCl2, EDTA 1 mM, 0.1% Triton X-100). Nuclei were separated from cytosol by centrifugation (10 min, 200 RCF, 4 °C) and discarded. Total protein content of the cytosol was determined by Qubit protein assay kit (Thermo Fisher Scientific, Waltham, MA, USA) according to the manufacturer’s instructions. After adding 10 μL of a solution containing 10 μg of total proteins present in the cytosols to the histone-coated strip wells (inhibitor solution), PARP1 activity was assayed by the HT Universal Colorimetric PARP Assay Kit in triplicate according to the manufacturer’s instructions. Briefly, 10 μL of cytosol extracts or 10 μL of the same buffer without proteins (control) were added to PARP1 substrate cocktail and into the strip wells and incubated for 60 min at room temperature. We measured the amount of incorporation of biotinylated poly (ADP-ribose) onto histone proteins in the strip well format by the addition of the TACS-Sapphire colorimetric substrate; the absorbance was read with a MultiSkan FC plate reader at 450 nm after stopping the reactions by adding 50 μL per well of 0.2 M HCl. Absorbance values were averaged and PARP1 activity was calculated from a standard curve.

### 2.14. Statistical Analysis

All statistical analyses were performed with the help of MedCalc for Windows, version 18.2.1 (MedCalc Software, Ostend, Belgium). Unless stated otherwise independent sample *t* test was used to compare the means of independent samples and two-sided *P*-values were considered significant if *P* < 0.05. When comparison of the means of multiple independent samples was appropriate, we performed ANOVA.

## 3. Results

### 3.1. Culture Conditions Modulate Glucose Consumption, Lactate Production and SHC3 Levels

We measured the rate of glucose consumption and lactate production of U-87 MG cells grown in vitro as three-dimensional multicellular tumor spheroids (MTS) or adherent to tissue culture plastic. Glucose consumption and lactate production rates were higher in MTS compared to adherent cultures (Figure 1A). We confirmed that p52SHC3 and p64SHC3, the two major protein isoforms encoded by the human *SHC3* gene, were higher in MTS [21] (Figure 1B,C) while the levels of the protein isoforms of the closely related gene *SHC1* were not increased (Figure 1B). Under our culture conditions the medium was totally depleted of glucose within 24 h. After that period, we found that U-87 MG cells could survive without medium replacement with no evidence of increased cell death for at least 5 days despite absence of glucose in the medium. We found that p52SHC3 and p64SHC3 also increased in U-87 MG cells growing in adherence after progressive depletion (by cell metabolism) or acute removal of glucose from the medium (Figure 1D,E). An inverse correlation between SHC3 levels and the level of glucose in the medium was also present in other glioblastoma cell lines and in primary cultures originally derived from the dissociation of human glioblastoma samples (Figure 1G, Figure S1A,B). Fetal calf serum was not essential for the effect since we found a comparable increase in SHC3 levels in the same cells maintained under serum free conditions.

The rates of glucose consumption and lactate production of glioblastoma cells, maintained for 120 h without medium replacement before the acute addition of fresh medium containing glucose (5 mM), were higher than the same rates in cells maintained under control conditions (medium replaced every 24 h) (Figure 1H). The level of SHC3 isoforms increased progressively after consumption of glucose in the medium (Figure 1I).

Growing cells without medium replacement does not only affect the level of glucose, which becomes negligible, but it is associated with a corresponding increase in lactate and other catabolites.

In order to evaluate if glucose exhaustion was the principal responsible for the increase of SHC3 in glioblastoma cells we added glucose (final concentration 5 mM) to cells growing in exhausted medium (Figure 1J,K and Figure S1C). After addition of glucose the level of SHC3 decreased and became comparable to those of cultures of the same cells where the medium was changed daily. Conversely, acute addition of lactate (final concentration 15 mM) to the same cells growing in fresh medium did not induce any increase in SHC3 (Figure 1J,K, Figure S1E). These results suggest that the lack of glucose and not the accumulation of lactate or other catabolites in the medium is mainly responsible for the increase in SHC3.

The effect of glucose starvation on SHC3 level is not species-specific since we obtained the same effect on C6 rat high-grade brain tumor cells. However, this effect appears restricted to tumor cells since in normal rat astrocytes prolonged glucose starvation did not induce either an increase of SHC3 or enhanced glucose uptake (Figure 1L,M).

### 3.2. Glucose Deprivation Enhances SHC3 Protein Stability

We compared the levels of SHC1 and SHC3 protein isoforms and the corresponding mRNAs by Western blotting and real time PCR in U-87 MG cells exposed to glucose depleted medium for 12 h and we found that the increase in SHC3 was not matched by an equal increase in *SHC3* mRNAs that remained stable (Figure 1N,O). We then studied, under the same experimental conditions, the stability of SHC3 protein isoforms by pulsed addition of L-azidohomoalanine (AHA) to the medium of U-87 MG cells. We harvested the cells and extracted the proteins at different time points after AHA addition and evaluated the AHA incorporated into proteins after conjugation to biotin by an alkyne-based reaction [37]. After immunoprecipitation of the protein extracts with an anti-SHC3 monoclonal antibody the level of biotin linked to SHC3 was higher in cells maintained in glucose deprived medium compared to cells maintained under control conditions (Figure 1P). This suggests that the increase in SHC3 isoforms induced by low glucose mainly depends on increased protein stability.

Further indication that glucose deprivation increases SHC3 levels predominantly through post-translational effects comes from HeLa cells transfected with a plasmid encoding the p52SHC3 cDNA under an artificial housekeeping promoter/enhancer complex (full length Moloney murine leukemia virus LTR/ immediate early hCMV enhancer) that is not sensitive to glucose [38]. In those cells, which do not express detectable endogenous SHC3, the level of transfected p52SHC3 was also increased by glucose deprivation (Figure 1Q, Figure S1H–J).

### 3.3. Increased SHC3 in Human Glioblastoma Cells Accelerates Glucose Consumption and Lactate Production by Increasing Glucose Uptake

**Figure 2:**
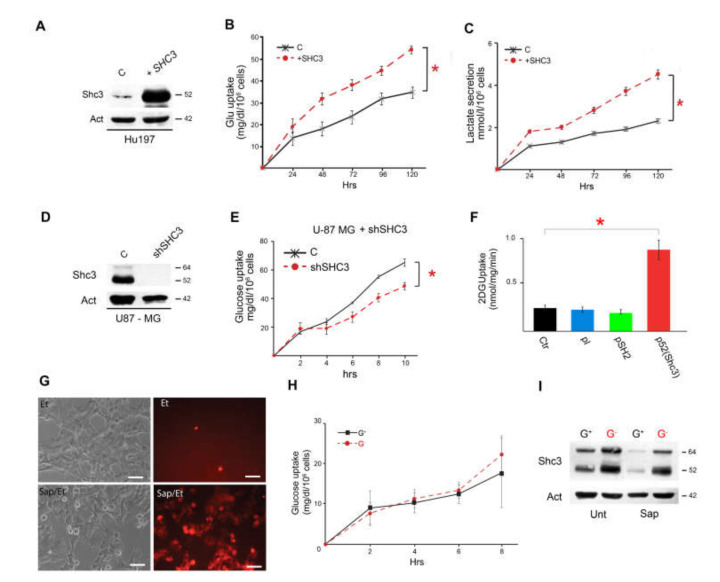
Increased SHC3 levels enhanced glucose consumption, lactate secretion and affected vesicle recycling. (**A**) Western blot using protein lysates from Hu197 after transfection with a control plasmid (**C**) or a plasmid encoding for p52SHC3 cDNA. (**B**) Rates of glucose uptake by Hu197 cells (black X) and by the same cells after transfection with cDNA encoding for p52SHC3 (red dots). Uptake of glucose in cells overexpressing *SHC3* cDNA is significantly increased. Data are means ± SD (at least n = 3 experiments, *: *P* < 0.05). (**C**) Rates of lactate secretion in the medium by Hu197 cells (black X) and by the same cells after transfection with cDNA encoding for p52SHC3 (red dots). Release of lactate in cells overexpressing *SHC3* cDNA is significantly increased. Data are means ± SD (at least n = 3 experiments, *: *P* < 0.05). (**D**) Western blot analysis using protein lysates from U-87 MG after transfection of a control plasmid or a plasmid encoding a shRNA targeting *SHC3* mRNA. (**E**) Time course of glucose consumption by U-87 MG 76 h after transfection with control (black) or shRNA targeting *SHC3* mRNA (red): shRNA transfection decreases significantly glucose uptake. (**F**) Rates of ^3^H-2DG uptake in Hu197 cells (Ctr) and after transfection of an empty plasmid (pl), or the same plasmid encoding for the SH2 domain of SHC3 (pSH2), or the same plasmid encoding for the entire p52SHC3. ^3^H-2DG uptake is enhanced only after p52SHC3 transfection. (**G**) Dark-field and fluorescent pictures of U-87 MG cells with and without membrane permeabilization with saponin. Red fluorescence results from the addition of ethidium bromide (EtBr), a membrane impermeant dye, whose presence inside the cells indicates membrane permeabilization. The cells that fluoresce in the absence of saponin (Ctr) are dying cells. Scale bar 10 mm. (**H**) Rates of glucose uptake in the presence of saponin. Before the addition of fresh medium containing saponin U-87 MG cells were maintained as shown in Figure 1D. Saponin cancelled the difference in glucose uptake between cells maintained with (G^+^) or without (G^−^) daily replacement of glucose. (**I**) Western blot analysis using protein lysates from U-87 MG growing according to Figure 1D without (Unt) or with Saponin treatment (Sap). The presence of saponin does not affect the increase in SHC3 levels induced by glucose deprivation (G^−^).

Under standard culture conditions, SHC3 is absent in HeLa cells (Figure 1Q) and barely detectable in Hu197 (Figure 1G, Figure 2A), while it is easily detected in U-87 MG cells (Figure 1E, Figure S1H). When we transfected those two glioblastoma cell lines with a plasmid encoding p52SHC3 cDNA (Figure 2A, Figure S1H), glucose consumption and lactate production increased (Figure 2B,C, Figure S1I,J). On the contrary, upon transfecting Hela cells with the same plasmid (Figure S1K), glucose consumption and lactate production did not increase (Figure S1L,M). Finally, reducing SHC3 levels in U-87 MG by transfection of a plasmid encoding a shRNA targeting *SHC3* mRNA (Figure 2D) resulted in decreased glucose consumption (Figure 2E). This effect was not present when we used a control shRNA.

We then measured the uptake of 2-deoxy-D-glucose (2-DG), a glucose analog that becomes trapped inside the cells after phosphorylation and it is not further metabolized, before and after transfection of U-87 MG and Hu197 cells with a plasmid encoding for p52SHC3 cDNA, or an empty plasmid, or a plasmid encoding only the SH2 domain of SHC3. We compared the results obtained by ^3^H labelled 2-DG and 2-deoxy-2-((7-nitro-2,1,3-benzoxadiazol-4-yl)amino)-D-glucose (2-NBDG) a fluorescent analog of 2-DG [39,40]. We found, using either substrate, a similar increase in glucose uptake in cells expressing p52SHC3 compared to their parental cell lines or to cells transfected with control plasmids (Figure 2F). An increase in 2-NBDG uptake was also present in U87-MG cells tested after glucose deprivation (Figure S2A), a condition that increase the intracellular concentration of SHC3. Treatment of U-87 MG cells with ritonavir, an inhibitor of multiple GLUT/SLC2A transporters [11], reduced the increase in 2-NBDG uptake and glucose extraction from the medium when SHC3 increased (Figure S2B), confirming that the increase in 2-NBDG uptake was linked to increased GLUT/SLC2A activity.

If the difference in glucose metabolism between glioblastoma cells grown under control conditions (medium changed every day) or stressed conditions (medium unchanged) was mainly due to increased glucose uptake in stressed cells, transient membrane permeabilization should abolish this difference. We permeabilized by saponin (25 μg/mL) [41] U-87 MG cells that were previously maintained in adherence for 96 h under control or stressed conditions (Figure 2G) and we compared the rates of glucose consumption after addition of fresh medium for 8 h, before visible signs of toxicity due to permeabilization became evident. We found that membrane permeabilization by saponin canceled the difference in the rate of glucose metabolism between the two conditions by increasing the rate of glycolysis in control cells (Figure 2H) without affecting the level of SHC3 (Figure 2I).

**Figure 3:**
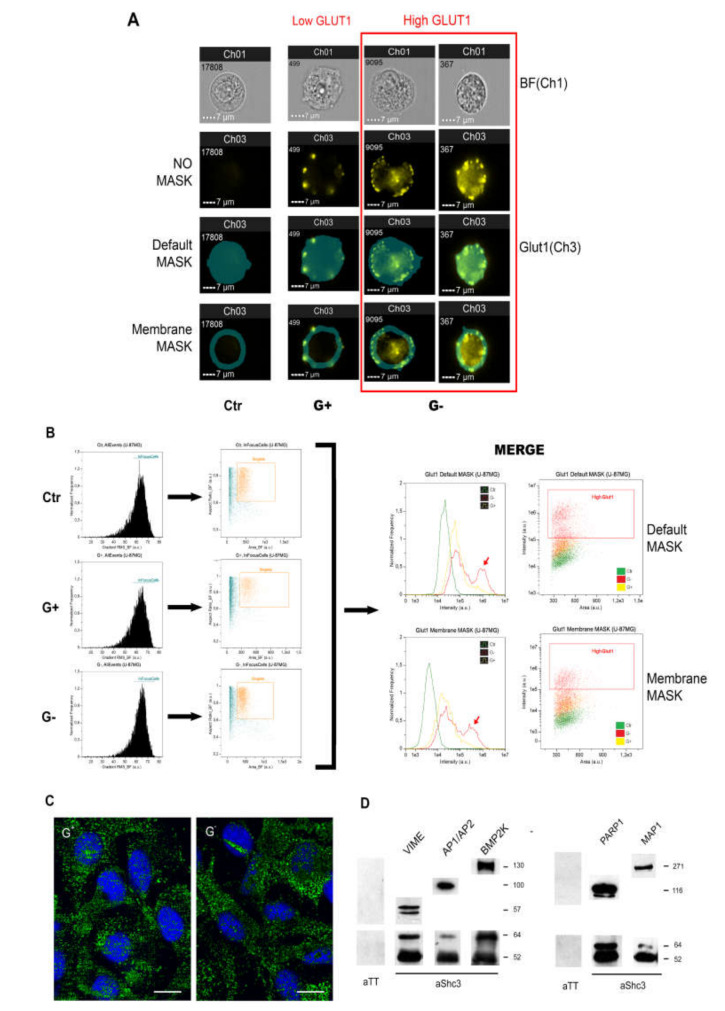
Glucose deprived cells have increased level of GLUT1 on the membrane and increased levels of SHC3 associated to cytoplasmic vesicles. (**A**) Living U87 MG cells were analyzed through an ImageStreamX MarkII using two channels: brightfield (Ch1) and fluorescence (Glut1Ch3). Four different representative cells are shown: the cell in column 1 is from control cells exposed only to the secondary phycoerythrin labeled antibody, the other columns contain cells exposed to anti-GLUT1 monoclonal followed by the secondary antibody. From above, row 1 brightfield images (BFCh1), row 2 immunofluorescence anti-GLUT1, row 3 and 4 illustrate the masks (cyan color) used for the differential quantification of whole cell (Default MASK) or membrane (Membrane MASK) immunofluorescence. “Membrane Mask” was defined by the following Boolean expression “*Object(M01,Ch1,Tight) And Not AdaptiveErode(M01,Ch01,80)*” created with the software Inspire (Amnis). Compared to the control secondary antibody (Ctr secondary antibody only), GLUT1 fluorescence (Glut1Ch3-) increases in cells maintained in glucose exhausted medium (G^−^) compared to cells maintained in normal glucose (G^+^). The cells contained in the red rectangle are examples of the cells that are enclosed in the red box shown in the scatter plot below (Figure 3B), showing high level of GLUT1 (High GLUT1). (**B**) Strategy of analysis. The different samples, control (Ctr), glucose rich (G^+^) and glucose deprived (G^−^) were analyzed by the following gating approach: “In Focus Cells” were identified based on the “Gradient Root Mean Square (RMS) Contrast Feature”, which captures in focus images of cells identified by high normalized pixel intensity gradient (RMS values) derived from the brightfield channel (BF); then, a scatter plot of the “Aspect Ratio Feature” versus brightfield “Area Feature” is used to identify single cells (singlets) from debris or cell clumps based on high aspect ratio and low area value; finally, the three samples were merged to evaluate GLUT1 expression variations in the histogram and a dot plot (Area vs Intensity). The GLUT1 “Intensity Feature” was evaluated on the two masks, Default and Membrane (A). Red arrows identified a peak in the immunofluorescence intensity that characterize glucose deprived (G^−^) cells. (**C**) A portion of SHC3 immunoreactivity in U-87 MG cells maintained in the presence (G^+^) or absence (G^−^) of glucose is associated with vesicles in the cytoplasm. Confocal images, scale bars: 10 μm. (**D**) Western blots of proteins immunoprecipitated by SHC3 monoclonal antibody (aSHC3) and identified by the specific antibodies as indicated above each lane. All these proteins were previously identified by MS. The negative control antibody recognized tetanus toxin (aTT); two membranes with cycles of probing, stripping and reprobing were used.

Glucose uptake in glioblastoma cells is mostly due to transporters of the GLUT/SLC2A superfamily and GLUT1, one of the constitutive glucose transporters, plays a prominent role [11]. We evaluated by image flow cytometry if in living U-87 MG cells GLUT1 increases under stressed conditions (medium unchanged) compared to the same cells maintained under control conditions. Living U-87MG cells with intact membranes were exposed to a monoclonal antibody against GLUT1 that was then revealed through a phycoerythrin labeled secondary antibody. All experiments were performed at 4 °C, as at this temperature most vesicle recycling is inhibited thus antibody binding in living cells should be mostly confined to GLUT1 molecules on the cell membrane. The intensity of anti-GLUT1 immunofluorescence increased in stressed U-87 MG cells compared to that of the same cells maintained under control conditions (ANOVA F-ratio 2391.632, *P* < 0.001). Increase in GLUT1 immunofluorescence in stressed U-87 MG cells was significant either when fluorescence intensity was measured considering the whole cell or when the measure was limited to the cell membrane (Figure 3A,B).

### 3.4. Multiple Proteins Involved in Vesicle Recycling Interact with SHC3

In U-87 MG cells, as in adult neurons [42], part of the immunoreactivity for SHC3 proteins is found in cytoplasmic and membrane bound vesicles (Figure 3C). We screened by liquid chromatography coupled with mass spectrometry (LC-MS) the proteins co-immunoprecipitating with SHC3 from whole cell extracts of U-87 MG maintained under normal and stressed conditions. We only considered proteins that were identified by LC-MS when the immunoprecipitating antibody was directed against SHC3 and were absent in controls (immunoprecipitated with antibody against tetanus toxin). We identified 694 proteins that were absent in control immunoprecipitations. Since activity of glucose transporters of the GLUT/SLC2A superfamily depends on membrane trafficking, we compared these proteins to the genes identified as involved in endocytosis by genome wide RNAi screening [43,44]. Of the 694 proteins we identified, 124 (17.87%) (Table S2) are involved in endocytosis according to the above studies. Collinet et al. in their study subdivided the effects on endocytosis in 14 phenotypic cluster groups and included SHC3 in cluster number 4 [43]. All phenotypic cluster groups were represented in the list of the proteins we identified; cluster number 4, the cluster containing SHC3, was not over represented and overall the frequency of proteins in each cluster group was not significantly different from the original (χ2, 10,877; DF, 13; *P* < 0.6211). We also found that eleven proteins that were previously associated with clathrin coated vesicles (CCV) [45] immunoprecipitated together with SHC3. Four of those eleven proteins were not comprised in the list of Collinet et al. [43] and were added (Table S1). Among the proteins involved with CCV the most frequently identified were all components of the adaptor protein complexes 1 and 2 (Ap1/Ap2).

In order to validate this association, we checked if Ap1/Ap2 complexes co-immunoprecipitated with SHC3 by Western blotting (Figure 3D). We also expressed in U-87 MG cells a fusion protein containing the rat AP2 alpha chain fused through its NH2 with eGFP [31] that was also immunoprecipitated together with SHC3, increasing our confidence in a bona fide interaction of SHC3 with Ap1/Ap2 complexes (Figure S2C). Among other proteins involved in vesicle traffic that co-immunoprecipitated with SHC3, we found microtubule associated protein 1A (MAP1A), BMP2K and vimentin (VIME). The presence of these proteins in the immunoprecipitates was also confirmed by Western blotting (Figure 3D).

In order to study if some of the interactions were influenced by post-translational modifications, we tested the ability of p52SHC3 fused to GST expressed in *Escherichia coli* to pull down SHC3 interacting proteins from U-87 MG whole cell extracts. Of the 128 proteins involved in endocytosis identified in the immunoprecipitation experiments only 5 were present in the pull-down experiments (Table S2).

### 3.5. Increased Glucose Uptake Is Linked to an Increase in Vesicles Trafficking

**Figure 4:**
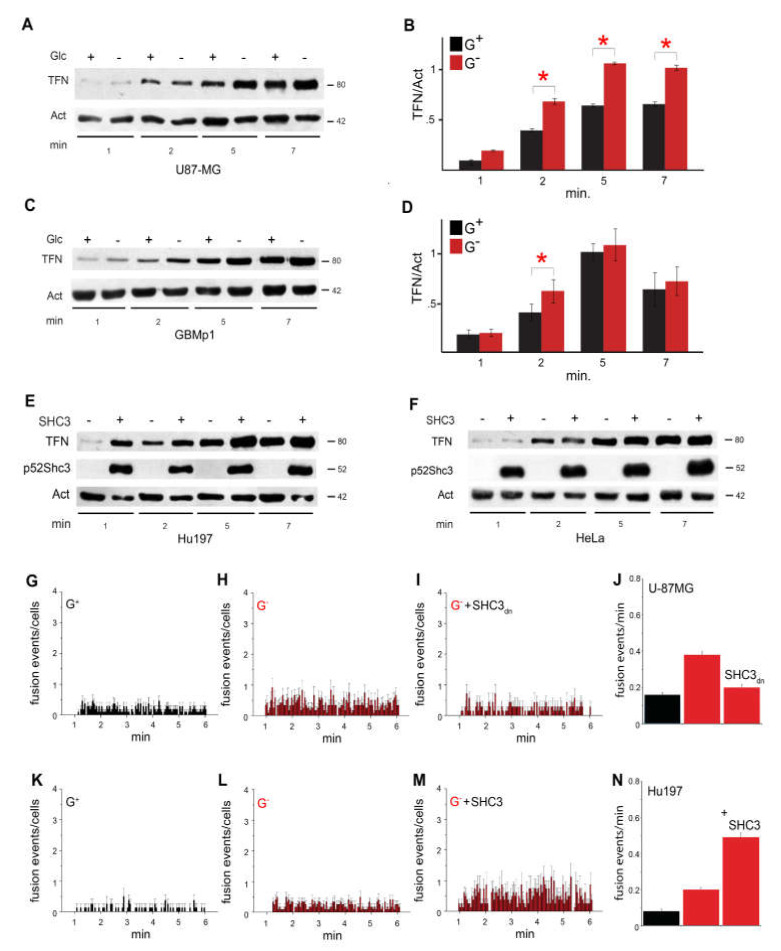
SHC3 modulates fusion of GLUTs containing vesicles to the membrane. (**A**,**C**) Western blot showing the time course of transferrin uptake by U-87 MG cells (**A**) and by short term cultures of glioblastoma-derived cells GBMP1 (**C**). Before addition of transferrin the cells were maintained in the presence (+) or absence of glucose (−) according to the scheme in Figure 1D. Tfn uptake increased significantly in cells maintained in the absence of glucose when SHC3 level increased. (**B**,**D**) Diagrams showing rates of transferrin uptake in U-87 MG and GBM-P1 derived from at least 3 experiments. Data as in (**A**,**C**). (**E**,**F**) Time course of transferrin uptake in Hu197 glioblastoma cells and Hela cells after transfection with p52SHC3 cDNA. Transferrin uptake was increased in Hu197 expressing p52SHC3 (+) compared to untrasfected ones (−), on the contrary the rate of transferrin uptake by HeLa cells expressing p52SHC3 was similar to that of untransfected cells. (**G**–**I**) U-87 MG cells, TIRF microscopy. Each bar represents the average frequency (3 s, at least 6 independent cells) of fusion to the membrane of vesicles containing GLUT4-eGFP; each cell was followed continuously for 6 min. The frequency of fusion increases in cells submitted to glucose deprivation (**H**) compared to cells maintained in the presence of glucose (**G**). This increase is inhibited by transfection in the same cells of a truncated version of p52SHC3 that has a dominant negative effect (**I**). Error bars: standard deviation. (**J**) Bar graph summarizing all the data for U-87-MG; column height: average; error bars: standard error of the mean. (**K**–**M**) Hu-197 cells, TIRF microscopy. Hu-197 cells express low levels of SHC3; same as before but at least 8 independent cells were followed for 6 min: frequency of fusion to the membrane of vesicles containing GLUT4-eGFP is only slightly increased after glucose deprivation (**L**), transfection of p52SHC3 cDNA enhances the increase in frequency of fusion events induced by glucose deprivation (**M**). Error bars: standard deviation. (**N**) As in Figure 4J but Hu-197 cells.

Glucose transporters of the GLUT/SLC2A are sensitive to changes in vesicle trafficking that modulate the number of GLUT/SLC2A present in the membrane. We studied Tfn receptor internalization, a commonly accepted marker of clathrin coated vesicles recycling [46], in U-87 MG cells under standard and stressed growth conditions. U-87 MG cells maintained for 6 days in the same medium after complete exhaustion of the glucose had significantly higher p52SHC3 levels and Tfn trafficking compared to the same cells maintained in the presence of glucose under standard conditions (Figure 4A,B). Similar results were also obtained with cells from a primary culture of glioblastoma (Figure 4C,D).

Human glioblastoma Hu197 cells maintained under standard conditions, but over-expressing exogenous p52SHC3, had also significantly increased levels of Tfn endocytosis compared to the same cells transfected with a control construct (Figure 4E, Figure S2D). Interestingly, Tfn trafficking did not increase in Hela cells expressing exogenous p52SHC3 when compared to control cells (Figure 4F, Figure S2E). We also confirmed the increase of the Tfn receptors trafficking in stressed U-87 MG and in SHC3-expressing Hu197 cells compared to controls by an alternative Tfn uptake assay (Figure S2F,G).

In order to verify that the increased vesicle trafficking induced by increased SHC3 levels affected also vesicles containing GLUT/SLC2As, we transfected U-87 MG cells with plasmids encoding either *GLUT3* or *GLUT4* cDNA fused to the enhanced green fluorescent protein gene, (*eGFP-GLUT3* or *eGFP-GLUT4*) and we evaluated in U-87 MG and Hu197 the dynamic of the vesicles containing these proteins by total internal reflection fluorescence (TIRF) under normal and glucose deprived conditions or with and without transfection of a plasmid encoding p52SHC3. Under our experimental conditions, glucose starvation in U-87 MG cells promoted a 2.0-fold increase of eGFP-GLUT4 fusion events in the TIRF evanescence field. This increase was inhibited by co-transfection of a plasmid encoding the PTB domain of SHC3 fused to the cyan variant of eGFP (Figure 4G,H,I,J) that is known to have a dominant negative effect [20,47].

A similar effect on vesicle trafficking was induced in Hu197 cells after transfection with a p52SHC3 encoding plasmid (Figure 4K,L,M,N).

### 3.6. SHC3 Is Associated with Vesicles That Contain GLUT/SLC2As and Other Proteins Co-Immunoprecipitating with SHC3

**Figure 5:**
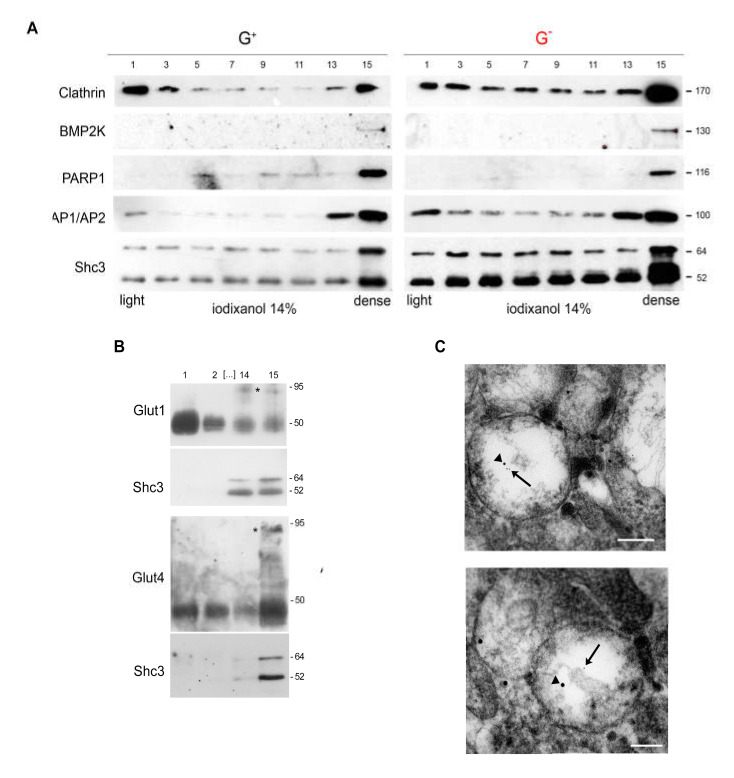
Glycosylated GLUTs co-fractionate with SHC3. (**A**) U-87 MG cells were maintained according to the scheme in Figure 1D, harvested, lysed and the low density microsomes (LDM) fraction separated by ultracentrifugation. The LDM fraction was further fractionated on self-generated iodixanol gradient and the fractions submitted to Western blotting analysis with the antibodies specified in the left column. SHC3, BMPK2, AP1/AP2 and PARP1 all peak in the densest fraction at a 14% iodixanol gradient. This is true both for cells deprived (G^−^) or not deprived (G^+^) of glucose. (**B**) Same as in 5A, U-87 MG cells were maintained under normal culture conditions and not deprived of glucose. The densest fractions of the 14% iodixanol gradient are enriched in Sch3 and glycosylated GLUT1 and GLUT4 (*) that are distinguishable from their unglycosylated forms by their higher apparent molecular weight. (**C**) Immunoelectron microscopy of vesicles contained in the LDM fraction. The presence of PARP1 (15 nm gold particles, arrowheads) and SHC3 (5 nm gold particles, arrows) in the same vesicles was revealed through secondary antibodies linked to the appropriate gold particles. Scale Barr: 200 nm.

The function of GLUT/SLC2A transporters is tightly regulated by changes in their subcellular localization. Transport of GLUT/SLC2As from the cytoplasm to the cell membrane has been linked both to clathrin-dependent and independent pathways [48]. In order to partially purify the vesicles containing SHC3 after U-87 MG cell lysis we removed the nuclei by low speed centrifugation and then separated the supernatant by ultracentrifugation into low density microsomes (LDM) and high density microsomes (HDM). We further fractionated the LDM fraction, which contains most of the vesicles associated to GLUT/SLC2A transporters, by ultracentrifugation in self-generated iodixanol gradient [49]. After fraction collection, we analyzed by Western blotting the distribution of the proteins of interest. We found that in self-generated gradients formed from 14% iodixanol, SHC3 concentration peaked in the denser fractions (Figure 5A) while in gradients from 30% iodixanol SHC3 remained confined to the lighter fractions (Figure S2H,I). At either iodixanol concentration, the peak SHC3 concentration is localized in the fraction containing APs and BMPK2 (Figure 5A), two proteins that co-immunoprecipitate with SHC3. We also found that the same fractions were enriched in PARP1, another protein we identified by MS as co-immunoprecipitating with SHC3 (Figure 5A and Supplementary Figure S2H,I).

Glucose transporters in a 14% iodixanol gradient show a double peak, one in the lower density fractions and the second in the high-density ones [49]. The second peak in the higher density fractions coincides with the peak of SHC3 and it is enriched in glycosylated GLUT/SLC2As (Figure 5B). Glycosylated GLUT/SLC2As are functionally mature transporters ready to be deployed and retained on the membrane for glucose uptake [48,49].

### 3.7. Immunoelectron Microscopy Confirms That There Are Vesicles Containing both SHC3 and PARP1

In order to confirm that SHC3 and PARP1 were associated to the same vesicles, we performed dual immunoelectron microscopic analysis of the vesicles present in the LDM fraction obtained from U-87 MG cells maintained under normal or stressed conditions. The presence of immunoreactive material for the two proteins was revealed through secondary antibodies linked to immunogold particles of different sizes. Examples of vesicles doubly positive for PARP1 (15 nm gold particles) and SHC3 (5 nm gold particles) are shown in Figure 5C.

### 3.8. SHC3 and PARP1 Interact Through the SHC3 PTB Domain

**Figure 6:**
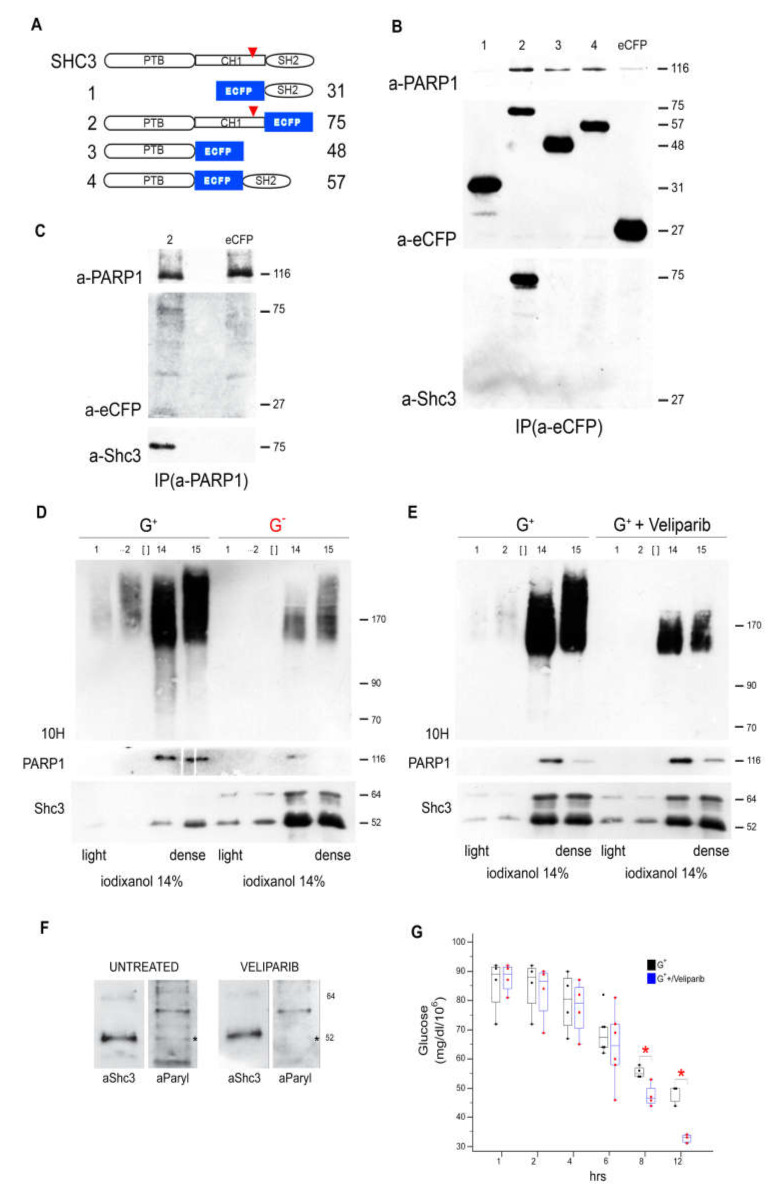
Glucose deprivation and veliparib affect PARylation of proteins in the vesicle containing SHC3 and PARP1 and increase glucose uptake by glioblastoma cells. (**A**) Schematic of the SHC3-eCFP constructs we used to identify the minimum requirement for the interaction of PARP1 with SHC3. (**B**) Western blot of the proteins immunoprecipitated with anti-eCFP monoclonal antibody from protein lysates of U-87 MG cells previously transfected with the constructs shown in Figure 6A. All constructs containing the PTB domain alone or in combination with the other domains of p52SHC3 were able to immunoprecipitate PARP1, while the SH2-eCFP fusion protein was not. Only construct 2 maintains the epitope (red arrowhead in Figure 6A) recognized by the anti-SHC3 antibody. The left column contains the names of the primary antibodies. (**C**) Western blot of the proteins immunoprecipitated with anti-PARP1 monoclonal antibody from protein lysates of U-87 MG cells previously transfected with construct 2 and eCFP. Only the construct containing the SHC3 PTB domain is immunoprecipitated together with PARP1 by the anti-PARP1 monoclonal antibody. The left column contains the names of the primary antibodies. (**D**) Western blot of U-87 MG protein lysates after fractionation on self-generated 14% iodixanol gradient. Distribution of PARylated proteins as demonstrated by monoclonal antibody 10H recognizing poly(ADP ribose) polymers of length equal or above 10, in absence of nuclear PARP1 activation. PARylated proteins peak in the fractions containing both SHC3 and PARP1. Glucose restriction (G^−^) according to the scheme in Figure 1D reduces PARylation of proteins in the same fractions containing SHC3 and PARP1. (**E**) As in D but comparing U-87 MG cells maintained under standard conditions in the presence or absence of veliparib (final concentration 1 μM); veliparib, similarly to glucose restriction, reduces PARylation of proteins in the same fractions containing SHC3 and PARP1. (**F**) Immunoprecipitation of protein lysates of U-87 MG cells; treatment of U-87 MG cells with veliparib, a specific inhibitor of PARP1, reduces p52SHC3 PARylation (*). Parylation was demonstrated by the use of a monoclonal antibody recognizing poly(ADP ribose) polymers of length equal or above 2. (**G**) Glucose uptake in U-87 MG maintained under standard conditions (black) or with the addition to the medium of veliparib (final concentration 1 μM). Box and whisker graph. Box represents values from the lower to the upper quartiles; middle line: median; vertical line connects minimum to maximum values excluding outliers. All experimental points from 4 separated experiments are plotted (one-way analysis of variance, * *P* < 0,001). Veliparib addition to the medium increases glucose uptake.

In order to map the region of interaction of SHC3 and PARP1, we transiently transfected U-87 MG cells with plasmids expressing combinations of the main SHC3 functional domains (i.e., SH2, PTB and CH1) fused at either the amino or carboxy-terminus of eCFP (Figure 6A). All fusion proteins were expressed with similar efficiency without acute toxicity (Figure 6B). Immunoprecipitation using an anti-eCFP antibody of U-87 MG cells extracts expressing these constructs showed that PARP1 was efficiently immunoprecipitated only when the fusion proteins contained the PTB domain while all other domains were dispensable and the fusion protein containing the SH2 domain alone was unable to bind PARP1 (Figure 6B). As expected, a SHC3 variant deleted only of the SH2 domain was also co-immunoprecipitated by an anti-PARP1 antibody (Figure 6C).

### 3.9. Parylated Proteins Concentrate in the Same Fractions Containing SHC3 and PARP1 and Treatment with Veliparib Reduces Parylation and Increases Glucose Uptake

We found by Western blotting that, without nuclear PARP1 activation, PARylated proteins concentrated in those fractions of the iodixanol gradient where PARP1 and SHC3 were maximal (Figure 6D). When the cells were maintained without replacing glucose in the medium the concentration of PARylated proteins decreased (Figure 6D) and the vesicles-associated PARP1 decreased (Figure 6D and Figure S2I). After immunoprecipitation we found that a fraction of SHC3 was also PARylated (Figure 6F).

**Figure 7:**
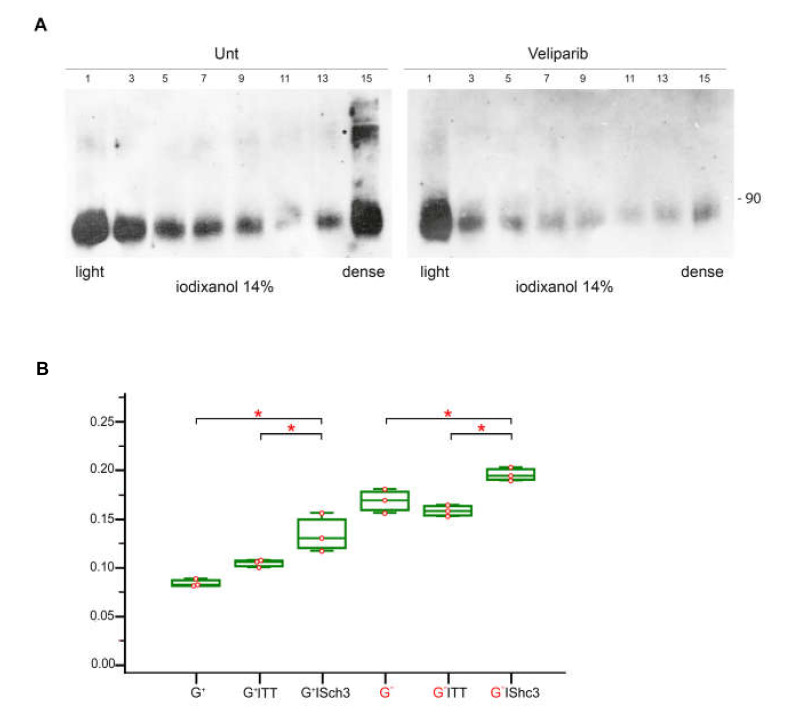
Veliparib induces a reduction of GLUTs cofractionated with SHC3 in vivo and immunodepletion of SHC3 decreases the inhibitory activity of cytosol of Hu197 cells on PARP1 activity in vitro. (**A**) Western blot using lysates of U-87 MG cells expressing a Glut4/GFP fusion protein, after fractionation on self-generated iodixanol gradient. In these cells, treatment with Veliparib induces a reduction in the level of the glycosylated Glut4/GFP (*) in the fractions of the self-generated 14% iodixanol gradient that contain both SHC3 and PARP1. (**B**) Box and whisker plot showing the effect of cytosol purified from Hu197 cells on the in vitro enzymatic activity of purified PARP1. Cytosols were extracted from Hu197 cells maintained as described in Figure 1D. G^+^: glucose changed every day; G^+^ITT: glucose changed every day, cytosol immunoprecipitated with antibody against tetanus toxin; G^−^: glucose exhausted medium; G^−^ITT: glucose exhausted medium, cytosol immunoprecipitated with antibody against tetanus toxin; G^-^ISHC3: glucose exhausted medium, cytosol immunoprecipitated with antibody against SHC3. Box represents values from the lower to the upper quartiles; middle line: median; vertical line connects minimum to maximum values excluding outliers; red circles: experimental points. Red * indicate significant difference (one-way ANOVA F-ratio 47.048, *P* < 0.001).

Veliparib is a specific inhibitor of PARP1 that is at least 2 orders of magnitude less active on tankyrase [50], another poly-ADP-ribosyltransferase that is associated to the insulin sensitive fraction of GLUT4/SLC2A4 containing vesicles [51]. After treatment with veliparib, PARylation of SHC3 decreased (Figure 6F) and PARylated proteins in those fractions typically enriched in PARP1 and SHC3 were reduced (Figure 6E). Moreover, after treatment with veliparib glucose uptake and lactate release in the medium increased (Figure 6G), while levels of glycosylated GLUT/SLC2As associated with the vesicles contained in the cytoplasm, decreased (Figure 7A).

Since these effects were also obtained by maintaining the cells without medium replacement when SHC3 protein increased, we tested if the cytosol of these cells had inhibitory effects on PARP1 activity of purified PARP in vitro. Using a colorimetric assay and purified PARP1 we found that in vitro the addition of 10 μg of total proteins purified from the cytosol of Hu197 human glioblastoma cells inhibited PARP1 activity (Figure 7B). This inhibition was partially reversed when the concentration of SHC3 in the cytosol was reduced by immunoabsorption with anti-SHC3 monoclonal antibody before adding the cytosol to the PARP1 activity assays. On the contrary, immunoabsorption of the cytosol with an antibody against tetanus toxin did not alter significantly the inhibitory activity of the SHC3-containing cytosol on PARP1 activity in vitro (Figure 7B).

## 4. Discussion

During glioblastoma and high-grade glioma growth in vivo, glucose may become rate limiting. Maintaining appropriate levels of intracellular glucose to support the aerobic glycolysis that sustains proliferation and survival of these tumors is thus of paramount importance for glioblastoma cells [12]. Multiple pathways are involved in controlling and enhancing glucose entrance in glioblastoma cells [11]. Our results suggest that in glioblastoma cells, an early response to glucose starvation is increasing SHC3 intracellular levels. SHC3 is a bifunctional phosphotyrosine binding protein [19] with two isoforms, p52SHC3 and p64SHC3, that were both increased in glioblastoma cells after glucose starvation. This increase in SHC3 content induced by lowered levels of glucose in the medium was also present in the absence of changes in cell density, another important factor that modulates the levels of SHC3 through FAK activation [18]. Addition of glucose but not lactate to the medium returned SHC3 to baseline in approximately 12 h. This was true both for glioblastoma cells in primary cultures and cell lines, but not for astrocytes, which are one of the closest normal counterparts of glioblastoma cells and are important players in brain energy production from circulating glucose [52,53].

Sudden addition of fresh medium containing 10 mEq of glucose to cells growing under standard conditions (baseline SHC3) or in exhausted medium (increased SHC3) demonstrated that the cells with higher SHC3 levels metabolize glucose to lactate at a rate that is higher than cells containing baseline SHC3 levels. This difference was abolished if the cells were pretreated with a shRNA targeting *SHC3* mRNA. The difference in the rate of glucose consumption was significantly reduced if the cell membrane was permeabilized by treatment with saponin, suggesting an effect on glucose uptake and not on the rate of glycolysis. Direct measurement of glucose internalization by the addition to the medium of ^3^H-DG or 2-NBDG, a fluorescent analog of 2-DG [39,40], confirmed that glucose internalization was increased in glioblastoma cells when levels of SHC3 were increased. Glucose uptake and lactate production increased in glioblastoma cells but not in HeLa cells after transfection of a plasmid encoding for p52SHC3.

Glucose uptake in glioblastoma cells is mainly dependent on transporters of the GLUT/SLC2A [6,11] superfamily. The membrane concentration of GLUT/SLC2As involved in glucose transport is tightly regulated and varies according to the type of the cell considered. Members of the GLUT/SLC2A are mainly located in intracellular membrane compartments and translocate to the cell membrane by clathrin-dependent and independent mechanisms [54].

We showed by IFCA that membrane concentration of GLUT1/SLC2A1, one of the glucose transporters present in high grade gliomas, was increased in living U-87 MG cells by glucose starvation compared to the same cells grown in presence of glucose. We also found that in glioblastoma cells where p52SHC3 levels were increased, internalization of transferrin, an accepted indicator of clathrin-dependent vesicles recycling [46], was significantly increased compared to the same cells with baseline p52SHC3.

Interestingly, when p52SHC3 was transfected in HeLa cells, a non-glial derived tumor cell line, we observed no increase in the uptake either of Tfn or of glucose, indicating that the effect of p52SHC3 increase is dependent on the cellular environment. The cellular contest where SHC3 acts is considered important for SHC3 function. For example, SHC3 activates PI-3K/Akt in neurons and glioblastoma cells [20] while in T lymphocytes it inhibits it [55].

We also confirmed an effect of increased level of SHC3 on vesicle trafficking by TIRF microscopy in U-87 MG cells. In those cells, the rate of fusion to the cell membrane of GLUT/SLC2As containing vesicles almost doubled when p52SHC3 was increased by glucose deprivation. This increase is indicative of an increased glucose-transporters translocation to the membrane [56,57] and is comparable to the effect of insulin stimulation on GLUT4/SLC2A4 translocation in insulin sensitive cells [56,57].

The results of co-immunoprecipitation and LC-MS experiments indicated that SHC3 interacts with at least 128 proteins known by RNA interference studies as involved in vesicle recycling and endocytosis [43,44]. Among the SHC3 interacting proteins involved in endocytosis that we most often isolated after immunoprecipitation and LC-MS there were the four adaptor related proteins (AP) that make up the heterotetrameric adaptor complex AP2, an essential component of endocytic membrane related clathrin-coated vesicles [26,58]. We also found the beta subunit of the AP1 complex that is an essential component of intracellular clathrin-coated vesicles [43,58]. Pull down of the α and ß isoforms of AP2 by SHC1, a protein closely related to SHC3, was previously demonstrated in vitro [59].

Among the most abundant proteins co-immunoprecipitating with SHC3, there were other proteins involved in vesicle recycling: BMP2K, microtubule associated protein 1A (MAP1A) and vimentin (VIM). BMP2K interacts with the AP2 complex bound to clathrin and the membrane [45] and stimulates binding of specific cargo proteins by phosphorylating AP2 [60]. Binding of MAP1A to AP2 [61] mediates both clathrin-coated vesicles (CCV) binding to microtubules and, after phosphorylation, the release of CCV from microtubules [61]. VIM is an intermediate filament protein typically associated with the epithelial to mesenchymal transition [62]. Moreover, VIM is involved with both clathrin-dependent and independent endocytosis [63] and may be necessary to locate the sodium-glucose transporter 1 (SGLT1) to rafts [64]. Fibroblasts lacking VIM were compromised in their vesicular zinc uptake, their organellar pH and their total and surface content of AP-3 cargoes [65]. Finally, a dynamic, flexible VIM-based network plays an important role in a number of signaling pathways [66]. We found that VIM not only co-immunoprecipitated with SHC3 but also bound to a column activated with purified human p52SHC3 expressed in bacteria, indicating that post-translational modifications of p52SHC3 were not necessary for VIM binding.

Altogether, the above results indicate that glucose deprivation in glioblastoma cells induces an increase in SHC3 levels, which modulates vesicle recycling leading to more GLUT/SLC2As on the membrane, thus resulting in an increase in glucose uptake. The interactions of SHC3 with adaptins and other proteins involved in clathrin-mediated exocytosis are at least partially responsible of the modulation of vesicle recycling by increased SHC3 levels.

We tried to further characterize the SHC3 fractions associated with vesicles by purification through ultracentrifugation on self-generated iodixanol gradients [49]. These experiments confirmed that SHC3 was enriched in the same fractions containing adaptins, GLUT/SLC2As and BMP2K. We also found that polyADP-ribose polymerase 1 (PARP1), a protein that co-immunoprecipitated with SHC3 but was not listed among those whose deficit interfered with endocytosis [43,44], was also enriched in the same fractions where SHC3 peaked. We confirmed the presence of SHC3 and PARP1 in the same vesicles by double immuno-electron microscopy. In absence of genomic stress PARP1 is mainly localized to the nucleus but to a lesser amount is also present in the cytoplasm [67,68]. PARP1 is a NAD-dependent poly-ADP-rybosiltransferase, as well as it is tankyrase-1, another poly-ADP-rybosiltransferase present in vesicles containing the fraction of GLUT/SLC2A4 whose recycling from the interior storage pool to the membrane is regulated in insulin sensitive cells by such hormone [69]. Among its multiple functions, PARP1 may interfere, mostly in an indirect way, with vesicle trafficking [70]. Moreover, treatment of cells derived from patients affected by cystic fibrosis with latonduine, an inhibitor of PARPs 1, 3 and 16, partially corrects the trafficking defect induced by the most common of cystic fibrosis conductance regulator, allowing the transfer of the mutated protein to the cell membrane [68,71].

We found that after iodixanol fractionation the SHC3 isoforms contained in the same fractions as PARP1, were PARylated, suggesting that PARP1 contained in the vesicles was active. Interestingly, in a proteome wide study of PARylated proteins present in human bone osteosarcoma-derived UOS2 cells that do not express SHC3, the closely related protein SHC1 was identified as PARP1 target [72].

**Figure 8:**
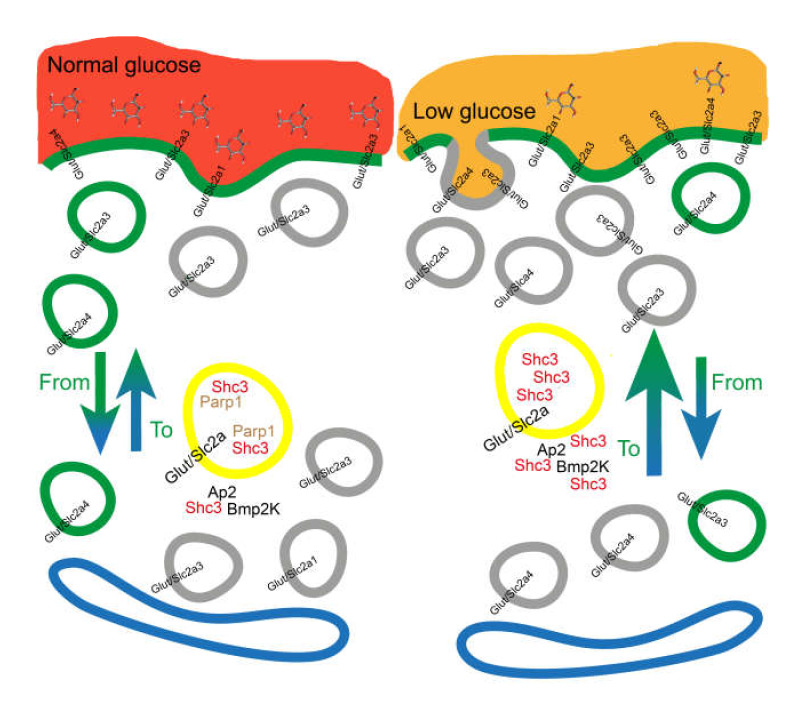
Glioblastoma cells maximize their glucose uptake by modulating glucose transporter trafficking. When glucose is freely available in the medium (normal glucose) trafficking of GLUT/SLCA2 containing vesicles to and from the membrane is balanced, when glucose is limited or absent (low glucose) vesicle trafficking becomes skewed towards the membrane. Some of the protein involved in this trafficking are Shc3, BMP2K, PARP1 and AP2. We found that in low glucose conditions SHC3 increases while vesicle associated PARP1 decreases.

After treatment of U-87 MG cells with veliparib, a specific inhibitor of PARP1 that is only a very week inhibitor of TNK1 [50], we measured an increase in glucose uptake and lactate production indicative of increased GLUT/SLC2A activity. Similarly, in insulin sensitive cells TNK1 inhibitors also increase glucose uptake [73]. Overall, our results suggest that lack of glucose in the medium induces an increase in SHC3 level that inhibits PARP1 activity and decreases its concentration in the vesicles associated with GLUT/SLC2A (Figure 8). This model is supported by our results showing that cytosol extracted from U87-MG cells inhibited the enzymatic activity of purified PARP1 in vitro. Accordingly, when we lowered the concentration of SHC3 in the cytosol by immunoabsorption with an anti-SHC3 monoclonal antibody, the inhibition of purified pPARP1 activity by these cytosol was significantly reduced.

We also found that in glioblastoma cells either glucose deprivation or veliparib treatment decreased protein PARylation in the fractions enriched in SHC3 and glycosylated GLUT/SLC2A. The same treatments also decreased the glycosylated fraction of GLUT4-eGFP fusion protein associated with the vesicles, further suggesting that in glioblastoma cells PARP1 has a role in GLUT/SLC2As trafficking. A role of PARP1 in glucose metabolism is also suggested by *Parp1*-knockout mice that are more susceptible to diet-induced insulin resistance and glucose intolerance compared with WT mice [74]. No data on glucose metabolism in *SHC3* knockout mice are available. However, the brain of those mice is more susceptible to oxygen and glucose deprivation [75]. Moreover, glycolysis is impaired in skeletal muscle and liver of knock-out mice for the closely related gene *SHC1* [25].

SHC3 contains two phospho-tyrosine binding domains that can mediate protein-protein interactions: a Src homology 2 domain (SH2) at the carboxy terminus and a phospho-tyrosine binding domain (PTB) at the amino terminus [19]. Our study of the interaction with PARP1 of multiple truncated fragments of SHC3 fused to eCFP indicated that the minimal requirement for co-immunoprecipitation was the presence of the PTB domain. Interestingly, PARP1 is a substrate of the tyrosine kinase cMet [76], which binds and phosphorylates SHC1 through an interaction also mediated by the PTB domain [77]. Binding of the PTB domain to proteins may be independent from the presence of phosphotyrosines, which may only modulate the stability of the interactions [77,78].

Glucose reduction inside all cells that, like glioblastomas, depend on this molecule for their metabolism, induces an increase in AMPK phosphorylation through activation of liver kinase B1 (LKB1) [79]. Increased phosphorylation of AMPK induced in glioblastoma by glucose starvation, stimulates phosphorylation by AMPK of octamer-binding transcription factor 1 (OCT1), inhibiting OCT1 ability to activate transcription of microRNA-451 (miR-451) [80]. MiR-451 is targeting for degradation the mRNAs coding for calcium binding protein 39 (CAB39), an essential binding partner of LKB1 [81]. By this mechanism, less miR-451 results in an increase in CAB39 that promotes phosphorylation of AMPK by LKB1 [80,81].

Accumulation of PARP1 in the cytoplasm through SHC3 interaction might decrease the level of PARP1 available in the nucleus for phosphorylation-independent activation of OCT1 [82]. Decreased OCT1 activation in the nucleus, among other effects, lowers the level of microRNA-451 in glioblastoma cells, resulting in enhanced AMPK activation with further stimulation of glucose uptake and metabolism [80].

## 5. Conclusions

In conclusion, our results suggest the existence of another pathway that, together with those already known [80], contributes to glioblastoma cells adaptation to sudden alterations of glucose in the microenvironment, ultimately resulting in glioblastoma survival.

Many of the proteins involved in this pathway represent druggable targets. No small molecule inhibitor of SHC3 is known, but, recently, an inhibitor of the closely related protein SHC1 has been described [83]. Moreover, other proteins in the pathway, for instance PARP1, have highly specific inhibitors that are currently tested alone or in combination with temozolamide against glioblastoma, both in experimental models and human subjects [84,85,86].

Our findings strengthen the evidence for a tight control of glucose transport in glioblastoma and suggest that combinations of drugs acting on GLUT/SLC2A [83] or interfering with their trafficking may be effective for glioblastoma treatment.

## Supplementary Materials

The following are available online at https://www.mdpi.com/2073-4409/9/5/1249/s1, Figure S1: Result of densitometry analyses and rates of glucose uptake and lactate production; Figure S2: Rate of glucose uptake, transferrin uptake, and result of separation of LDM fraction on 30% iodixanol gradient; Table S1: Key resource table; Table S2: List of proteins co-immunoprecipitating with SHC3 that are involved in endocytosis or associated with clathrin-coated vesicles.
