# Supplementary material for: A New Pathway Promotes Adaptation of Human Glioblastoma Cells to Glucose Starvation
Source: Cells. 2020 May 18;9(5):1249. doi: 10.3390/cells9051249 (PMC7290719; doi:10.3390/cells9051249)
Supplement: Supplementary file 1 [file cells-09-01249-s001.zip › Supplemental Materials/Captions.docx]

**Supplementary Figures and Table captions:**

**Supplementary Figure 1.** (**A**) Densitometry analysis of western blots (anti SHC3 monoclonal antibody) of total protein extracts of human glioblastoma cell line Hu197 maintained as indicated in D; G^+^ (black): medium changed daily, G^-^ (red) medium unchanged. Data are means ± SD (at least n = 3 experiments, *= p < 0.05), a.u. optical density in arbitrary units. (**B**) Same as in Supplementary Figure 1A but protein extracts from primary cultures of human glioblastoma GBM-P1, 2 and 3. (**C**) Same as in Supplementary Figure 1A but protein extracts from U-87 MG maintained as indicated in Figure 1L with the addition of 10 mmol/l of glucose (+G) to the medium 8 hours before harvesting the cells. Glucose significantly decreased the level of SHC3 suggesting that lack of glucose and not other molecules accumulating in the exhausted medium is mainly responsible for the increase in SHC3. (**D**) Same as in Supplementary Figure 1C but protein extracts from primary cultures of human glioblastoma GBM-P1. (**E**) Same as in Supplementary Figure 1C but with the addition to the U-87 MG cells of 10 mmol/l of lactate (+L). (**F**) Densitometry analysis of western blots of proteins immunoprecipitated with anti SHC3 monoclonal antibody from U-87 MG cells pulse-labeled with AHA and than maintained as outlined in Figure 1D. Colors, abbreviations and data as in Supplementary Figure 1A. (**G**) Densitometry analysis of western blots of protein lysates of HeLa cells (UNT) and HeLa cells (+SHC3) after transfection with a plasmid encoding for the cDNA of p52SHC3. (**H**) Western blots using protein lysates from U-87 MG cells after transfection with a control plasmid (C) or a plasmid encoding for the cDNA of p52SHC3 (+SHC3). Antibodies indicated on the left. (**I**, **J**) Rates of glucose uptake and lactate production in U-87 MG cells transfected with a control plasmid (C) or a plasmid encoding for p52SHC3 cDNA (+SHC3). After transfection the rate of glucose uptake and lactate production are increased compared to controls. Data as in Supplementary Figure 1A. (**K**) as in Supplementary Figure 1A but extracts from Hela cells. (**L**, **M**) Rates of glucose uptake and lactate production in HeLa cells transfected with a control plasmid (C) or a plasmid encoding for p52SHC3 cDNA (+SHC3). In HeLa cells there were no changes in glucose uptake and lactate production after transfection. Data as in Supplementary Figure 1A.

**Supplementary Figure 2.** (**A**) Rate of NBDG uptake in U-87 MG cells maintained according to the scheme in Figure 1D is increased in glucose deprived cells (G^-^, red) compared to controls (G^+^, black). Data are means ± SD (at least n = 3 experiments, *= p < 0.05). (**B**) Increased rates of glucose uptake in U-87 MG maintained without daily change in glucose containing medium (black) is reversed by treatment with ritonavir a specific inhibitor of GLUT/SLC2A (yellow, Rit). Data are means ± SD (at least n = 3 experiments, *= p < 0.05). (**C**) Western blot of immunoprecipitation of protein lysate of U-87 MG cells previously transfected with a control plasmid encoding for eGFP or a plasmid expressing the Ap1 protein fused to eGFP. The immunoprecipitating antibody recognized SHC3. (**D** ) Western blot. Time course of transferrin uptake measured by an alternative method in U-87 MG cells maintained according to the scheme in Figure 1D. SHC3 increase induced by lack of glucose in the medium (G^-^ red) is associated to an increase in transferrin uptake. (**E**) Western blot. Transferrin uptake measured by an alternative method, was increased in Hu197 cells transfected with p52SHC3 cDNA (red) compared to cells transfected with empty plasmid (black). (**H**, **I**) Western blot. U-87 MG cells were maintained according to the scheme in Figure 1D, after 129 hours the cells were harvested lysed and the LDM fraction separated by ultracentrifugation. The LDM fraction was further fractionated by centrifugation on self-generated 30% iodixanol gradient. At this density SHC3 and PARP1 peak in the lightest fraction instead of the densest as in 14% iodixanol gradient. Cells maintained in chronic absence of glucose (I) have less PARP1 in the vesicles compared to those maintained changing daily the medium (H).

**Supplementary table 1 (Table S1):** Key resource table.

**Supplementary Table 2 (Table S2)**: the table contains 128 proteins, identified by LC-MS after immunoprecipitation with anti SHC3 monoclonal antibody, that are involved in endocytosis [46,47] or associated with clathrin coated vesicles (CCV) [48].

For each protein the UniProt entry, Protein name and corresponding gene name are indicated **Collinet's cluster**: phenotypic cluster groups as indicated in Collinet et al., 2010 [46]. Proteins without a cluster number are not present in Collinet's list but were identified as associated with CCV [48].

**Pull down**: Y indicates proteins in a cellular extract that were pulled down using p52SHC3-GST expressed in *E. coli*.

**Out of 17**: Frequency of identification of the protein by LC-MS considering all 17 CoIP experiments.

**Hits tot**:  Average value of the SEQUEST protein hits (i.e. number of MS/MS spectra associated to the protein) calculated for each protein using its identification frequency “out of 17”.

**Score tot**: Average value of the SEQUEST protein score calculated for each protein using its identification frequency “out of 17”.

**Out of 10 G+**: Frequency of identification calculated for each protein considering only the 10 CoIP experiment done on U-87 MG cells growing in glucose rich medium (medium changed every day).

**Hits G^+^**: as in "Hits tot" but calculated only for experiments with cells growing in glucose rich medium.

**Score G^+^:** as in "Score tot" but calculated only for experiments with cells growing in glucose rich medium.

**Out of 7 G^-^**: frequency of identification calculated for each protein considering only the 7 CoIP experiment done on U-87 MG cells growing in glucose poor medium (medium unchanged).

**Hits G^-^**: as in "Hits tot" but calculated only for experiments with cells growing in glucose poor medium.

**Score G^-^**: as in "Score tot" but calculated only for experiments with cells growing in glucose poor medium.
